# Molecular domino reactor built by automated modular synthesis for cancer treatment

**DOI:** 10.7150/thno.43581

**Published:** 2020-03-04

**Authors:** Yu Yang, Jiaxuan He, Wenjun Zhu, Xiaoshu Pan, Hoda Safari Yazd, Cheng Cui, Lu Yang, Xiaowei Li, Long Li, Liang Cheng, Liangzhu Feng, Ruowen Wang, Zhuang Liu, Meiwan Chen, Weihong Tan

**Affiliations:** 1 Institute of Molecular Medicine (IMM), Renji Hospital, State Key Laboratory of Oncogenes and Related Genes, Shanghai Jiao Tong University School of Medicine, and College of Chemistry and Chemical Engineering, Shanghai Jiao Tong University, Shanghai 200240, China; 2 State Key Laboratory of Quality Research in Chinese Medicine, Institute of Chinese Medical Sciences, University of Macau, Taipa, Macau, China; 3 Molecular Science and Biomedicine Laboratory (MBL), State Key Laboratory of Chemo/Biosensing and Chemometrics, College of Chemistry and Chemical Engineering, College of Biology, Aptamer Engineering Center of Hunan Province, Hunan University Changsha, Hunan, 410082, China; 4 Department of Chemistry, Department of Physiology and Functional Genomics, Center for Research at Bio/Nano Interface, Health Cancer Center, UF Genetics Institute, McKnight Brain Institute, University of Florida, Gainesville, Florida 32611-7200, USA; 5 Institute of Functional Nano & Soft Materials Laboratory (FUNSOM), Soochow University, Suzhou, Jiangsu 215123, China

**Keywords:** Automated modular synthesis, Molecular domino reactor, Molecular “elements”, Tumor microenvironment (TME), Photodynamic therapy

## Abstract

**Rationale**: A cascade, or domino, reaction consists of two, or more, consecutive reactions such that subsequent reactions occur only if some chemical functionality has first been established in the prior step. However, while construction of predesigned and desired molecular domino reactors in a tailored manner is a valuable endeavor, it is still challenging.

**Methods**: To address this challenge, we herein report an aptamer-based photodynamic domino reactor built through automated modular synthesis. The engineering of this reactor takes advantage of the well-established solid-phase synthesis platform to incorporate a photosensitizer into G-quadruplex/ hemin DNAzyme at the molecular level.

**Results**: As a proof of concept, our photodynamic domino reactor, termed AS1411/hemin- pyrochlorophyll A, achieves *in vivo* photodynamic domino reaction for efficient cancer treatment by using a high concentration of hydrogen peroxide (H_2_O_2_) in the tumor microenvironment (TME) to produce O_2_, followed by consecutive generation of singlet oxygen (^1^O_2_) using the pre-produced O_2_. More specifically, phosphoramidite PA (pyrochlorophyll A) is coupled to aptamer AS1411 to form AS1411-PA ApDC able to simultaneously perform *in vivo* targeted imaging and photodynamic therapy (PDT). The insertion of hemin into the AS1411 G-quadruplex was demonstrated to alleviate tumor hypoxia by decomposition of H_2_O_2_ to produce O_2_. This was followed by the generation of ^1^O_2_ by PA to trigger cascading amplified PDT.

**Conclusion**: Therefore, this study provides a general strategy for building an aptamer-based molecular domino reactor through automated modular synthesis. By proof of concept, we further demonstrate a novel method of achieving enhanced PDT, as well as alleviating TME hypoxia at the molecular level.

## Introduction

A cascade, or domino, reaction is a consecutive series of reactions in which subsequent reactions depend on the previous establishment of chemical functionality 1, 2. In nature, domino reactions 3, 4, including enzyme dominos 5-7, occur in spatially constrained environments. It is in biological environments that chemical transformations occur in the biological system via the cellular metabolic pathway, enabling changes in the physiological features of living organisms. However, the development of a synthetic reactor requires that it be spatially confined to a structure that will allow for the generation of a high local concentration of intermediates for improved efficiency of biocatalytic dominos, and this remains a scientifically challenging task 7-9.

To address this challenge, we turn to aptamers 10-13, also called “chemical antibodies”. Aptamers are single-stranded DNA or RNA oligonucleotides that can specifically recognize targets with high affinity and thus have been studied for use in targeted cancer therapy 14-17. DNA is a well-understood material. With specificity of interactions between complementary base pairs, it is very suitable for building structures that must adhere to rigorous spatial parameters. (e.g., specificity of the interactions between complementary base pairs), that is appropriate to build the predesigned and desired structure of spatial organization. Solid-phase synthesis 18-20 enables the synthesis of sequence-predesigned DNA or RNA macromolecules through covalent bonding of molecules to a solid support material, allowing step-by-step synthesis in a single reaction vessel using selective protecting group chemistry. The use of phosphoramidite building blocks (A, T, U, C, and G) as base “elements” is finally exploited in this efficient and simple system 21, 22. The merits of this method include efficiency, simplicity and speed. Inspired by this technology, we and others have developed various artificial bases to expand the functions and applications of DNA molecules based on their unique properties 18, 23-25. For instance, our group and others have recently exploited the artificial Z P bases 26 to construct Hachimoji DNA^27^ and aptamer-drug conjugates (ApDCs)^23^ via solid-phase synthesis. Despite these achievements, exploitation of new molecular “elements” to construct an aptamer-based molecular domino reactor remains, as noted above, a daunting task.

Hypoxia and high hydrogen peroxide levels, as two unique physiological features of the TME, drive the Warburg effect, in which cancer cells tend to favor metabolism via glycolysis, even in aerobic conditions, rather than the much more efficient oxidative phosphorylation pathway, which is preferred by most other cells of the body 28. Thus, tumor growth and metastasis are, in turn, promoted and lead to increased resistance to cancer therapy, especially photodynamic therapy (PDT) using singlet oxygen (^1^O_2_) 29-35. Recently, smart nanotheranostic approaches have achieved targeted delivery and overcome tumor hypoxia 36-40, but many of those methods require sophisticated designs and complicated fabrication. Therefore, clinically translatable applications still call for automated modular synthesis of ApDCs with spatial controllability to act as a molecular reactor for *in vivo* cascading PDT. As designed in this work, a high level of H_2_O_2_ in the TME is used to produce O_2_ for subsequent generation of ^1^O_2_. Meanwhile, owing to the reduction of H_2_O_2_ and production of O_2_ during the domino reaction, multiple parameters of TME resistant to cancer treatment are eliminated.

**Figure 1:**
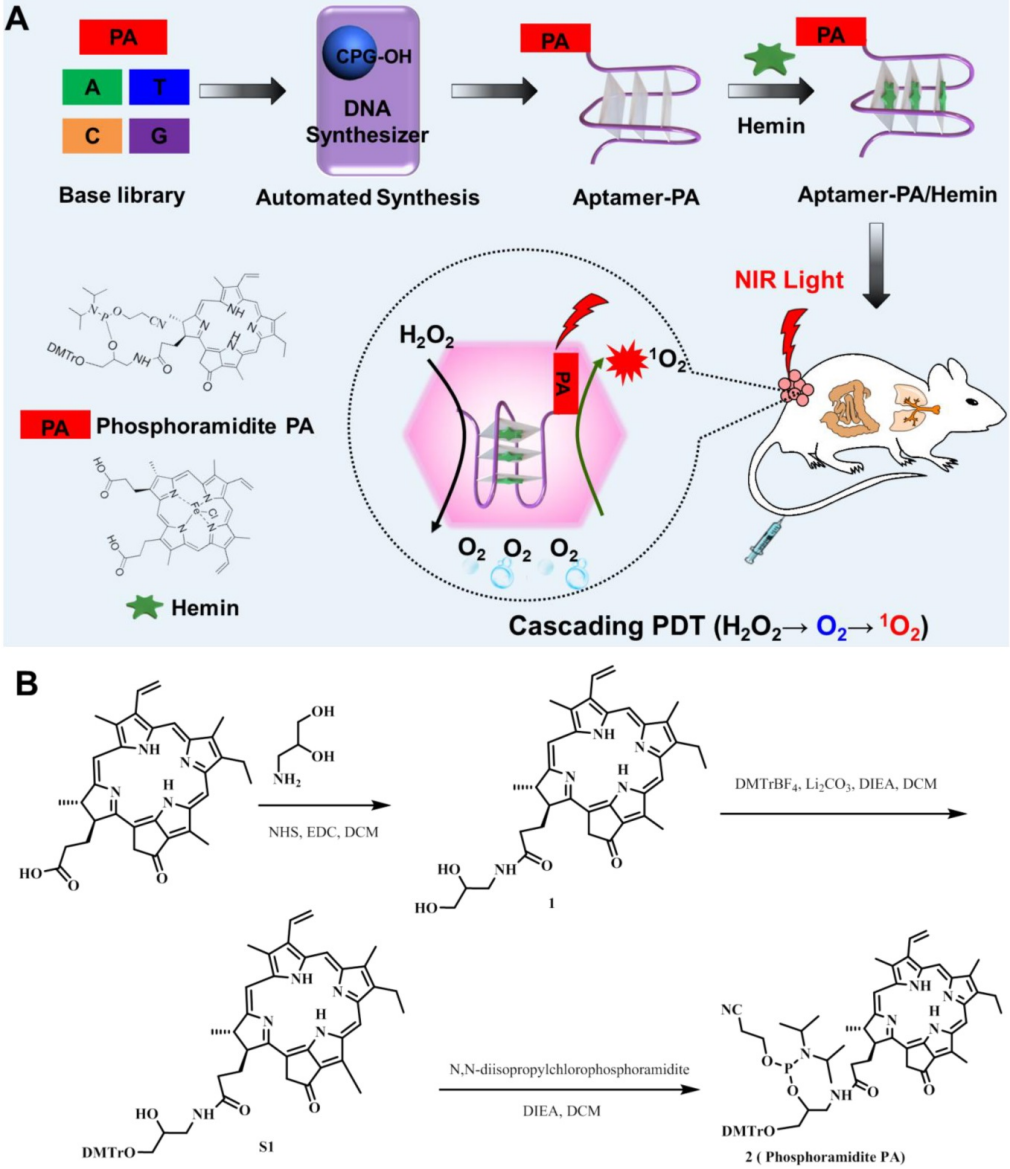
(A) Scheme illustrating automated and modular synthesis of AS1411/hemin-PA ApDC as a molecular domino reactor and its application for cascading amplified photodynamic therapy. Inside the tumor microenvironment, AS1411/hemin-PA, as a photodynamic domino reactor, can catalyze two consecutive reactions, i.e., decomposition of inherent H_2_O_2_ to produce O_2_ and utilization of produced O_2_ to generate ^1^O_2_ for cascading amplified PDT. (B) Synthetic route of phosphoramidite PA as a module for DNA solid-phase synthesis. Pyrochlorophyll-A (PA), a photosensitizer, was linked with solid-phase synthesis functionalities to form modular phosphoramidite-PA for automated solid-phase synthesis of AS1411-PA ApDC. Subsequently, hemin, an iron-containing porphyrin, was inserted into the G-rich region of AS1411 to form AS1411/hemin-PA with G-quadruplex/hemin DNAzyme structure.

Our problem in this paper was to construct a synthetic reactor capable of executing this task, and we herein report the development of an ApDC, termed pyrochlorophyll-A^41^ (PA)-aptamer/hemin (AS1411/hemin-PA), a molecular conjugate designed to decompose inherent H_2_O_2_ to produce O_2_ and then utilize the produced O_2_ to generate ^1^O_2_ in a confined DNA structure. As shown in **Figure 1A**, we first synthesized phosphoramidite-PA containing solid-phase synthesis functionalities and pyrochlorophyll-A (PA), an efficient photosensitizer widely used in PDT. Subsequently, phosphoramidite PA was coupled on the aptamer AS1411 42, a G-rich oligonucleotide sequence which specifically recognizes the highly expressed nucleolin on the surface of cancer cell membranes 43, using ploy(T) base as a linker, thus forming AS1411-PA ApDC for *in vivo* targeted imaging and PDT. Finally, hemin, an iron-containing porphyrin, was inserted into the G-rich region to form G-quadruplex/hemin DNAzyme, which can decompose H_2_O_2_ intrinsic to the TME into oxygen *via* the Haber-Weiss reaction 39, 44. Subsequently, the generated oxygen can be employed for PA under NIR light irradiation to produce ^1^O_2_, achieving a photodynamic domino reaction for cancer treatment. As evaluated by *in vitro* and* in vivo* studies, AS1411/ hemin-PA can be regarded as an efficient PDT agent with highly selective and specific recognition of cancer cells, alleviating tumor hypoxia resistance by decomposing H_2_O_2_ into O_2_ for enhanced PDT.

## Results and Discussion

**Figure 2:**
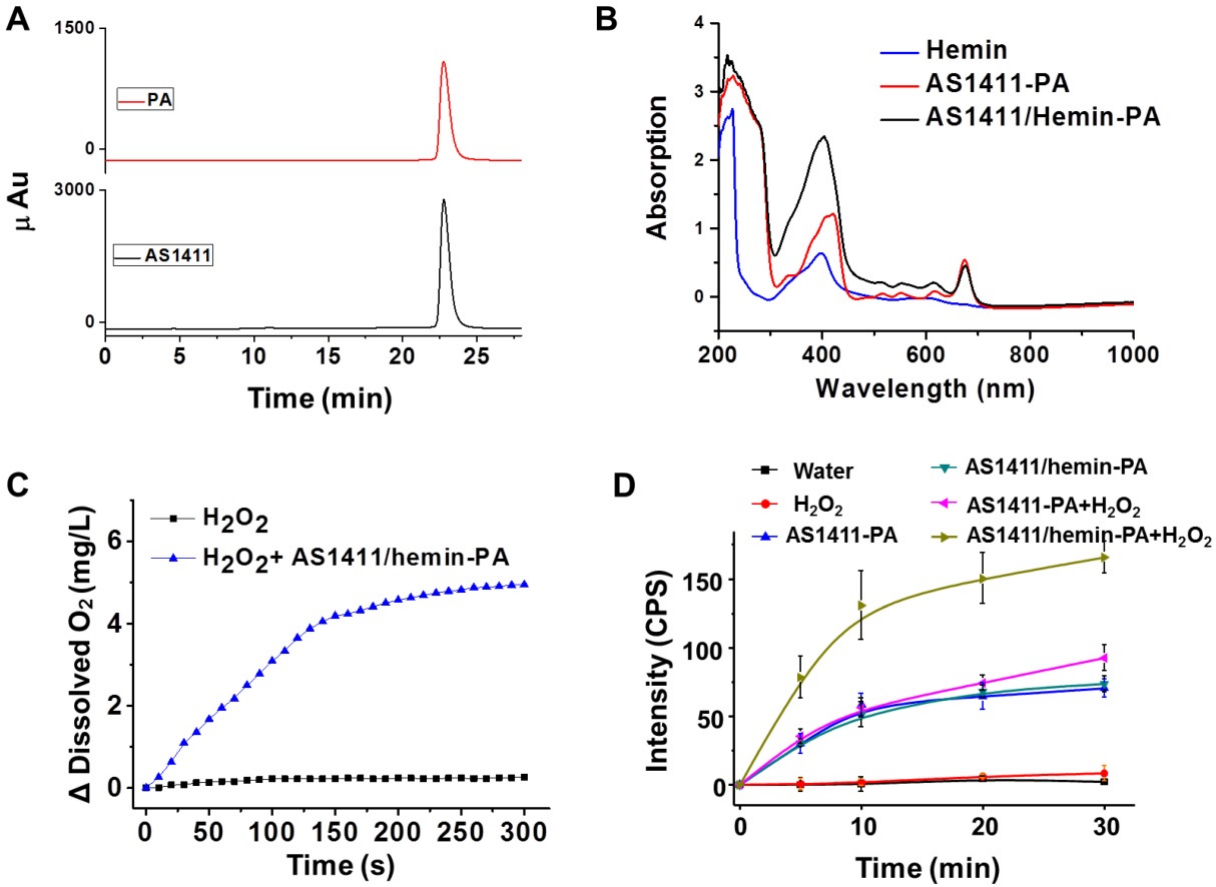
Characterization of AS1411/hemin-PA. (A) HPLC chromatogram of AS1411-PA conjugate. (B) UV-vis-NIR spectra of hemin, AS1411-PA, and AS1411/hemin-PA. (C) Oxygen generation in hydrogen peroxide solution with or without addition of AS1411/hemin-PA at room temperature. (D) Singlet oxygen generation, as determined by the increased SOSG fluorescence of AS1411-PA and AS1411/hemin-PA with or without hydrogen peroxide under 670-nm NIR light irradiation.

To achieve automated modular synthesis of ApDCs, therapeutic phosphoramidite PA module, as a coupling agent, was first synthesized. As shown in **Figure 1B**, 3-amino-1,2-propanediol was used to acylate pyrochlorophyll A to obtain compound 1 as a black solid in 82% yield. Next, compound 1 and N,N- diisopropylchlorophosphoramidite were converted into compound 2 (phosphoramidite PA) in 62% yield in two steps following standard protocols. The prepared modular phosphoramidite PA was then coupled on the AS1411 aptamer using poly(T) base as a linker, via solid-phase synthesis technology to form AS1411-PA ApDC **(Figure S1-S5)**. After automated synthesis, AS1411-PA was purified by HPLC and showed the typical PA absorption band at 680 nm, indicating successful coupling of phosphoramidite PA to AS1411 aptamer **(Figure 2A, 2B)**. Next, hemin was chosen for insertion into the AS1411 G- quadruplex structure to form the AS1411/hemin-PA conjugate, demonstrating typical UV-vis-NIR absorption peaks of hemin and PA at ~ 400 nm and 680 nm, respectively **(Figure 2B)**. Dynamic light scattering (DLS) measurement showed that the hydrodynamic sizes of AS1411-PA and AS1411/ hemin-PA were around 10 nm **(Figure S6)**, the latter of which exhibited excellent stability in physiological environment** (Figure S7)**.

It is well known that tumor hypoxia in the TME is critical to tumor angiogenesis and metastasis and that it leads to increased resistance to cancer treatment, especially oxygen-driven PDT 37, 45, 46. Ferric chloride hemin, which endows G-quadruplex with DNA enzyme capacity, can decompose H_2_O_2_ in the solid tumor into oxygen via the Haber-Weiss reaction, which can then overcome the multiple factors of TME resistance for improved PDT. As shown in **Figure 2C**, domino the concentration of oxygen in the hydrogen peroxide solution was significantly increased after addition of AS1411/hemin-PA, indicating that our AS1411/hemin-PA could catalytically decompose hydrogen peroxide to oxygen. Subsequently, the produced O_2_ could be used as a reactant to generate ^1^O_2_ from the PA under 670-nm irradiation*.* As shown in **Figure 2D**, without photosensitizer PA, both water and H_2_O_2_ cannot produce ^1^O_2_ after irradiation by 670-nm light. Since free oxygen in the atmosphere is dissolved in water, AS1411/hemin-PA and AS1411-PA showed moderate light-induced ^1^O_2_ generation under NIR light. Interestingly, in the presence of H_2_O_2_, AS1411-PA showed limited improvement of ^1^O_2_ generation under NIR irradiation, whereas light-induced ^1^O_2_ production by AS1411/hemin-PA was significantly improved. This is attributed to AS1411/hemin-PA, which can act as a molecular reactor to achieve cascading photodynamic reaction by utilizing H_2_O_2_ to produce O_2_, which is further utilized by PA exposure to NIR light to produce ^1^O_2_. Owing to these consecutive reactions, AS1411/hemin-PA can be used as an ideal PDT agent for amplified therapeutic PDT efficacy.

**Figure 3:**
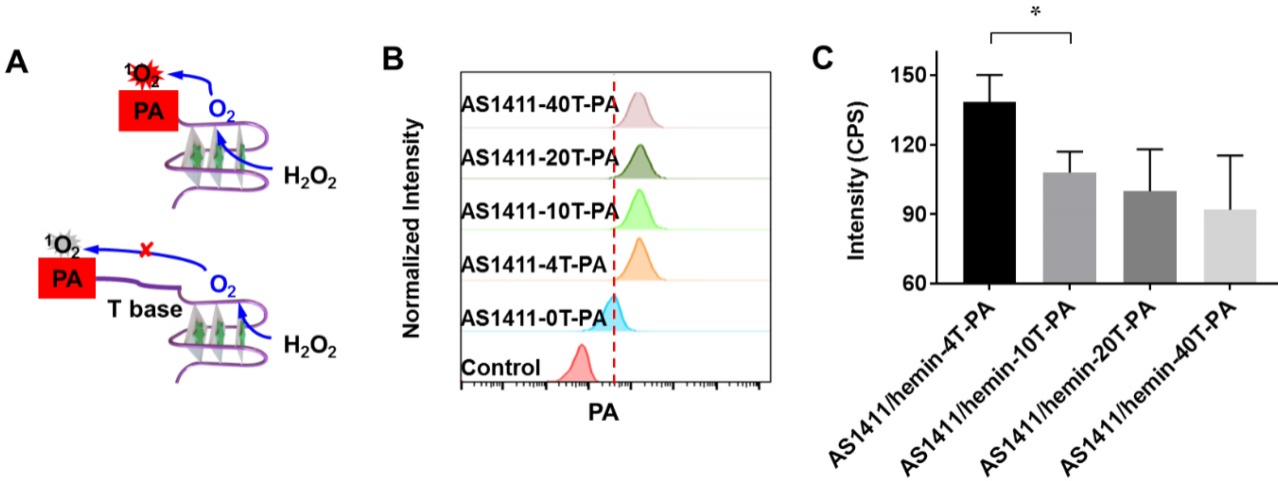
(A) Scheme of aptamer-based molecular domino reactor to generate ^1^O_2_. (B) Flow cytometry assay showing the binding affinities of MCF-7 cell after incubation of AS1411-0T-PA, AS1411-4T-PA, AS1411-10T-PA, AS1411-20T-PA, AS1411-40T-PA. (C) Singlet oxygen generation, as determined by the increased SOSG fluorescence, of AS1411/hemin-4T-PA, AS1411/hemin-10T-PA, AS1411/hemin-20T-PA, and AS1411/hemin-40T-PA in the presence of hydrogen peroxide under 670-nm NIR light irradiation for 10 min. P values in (C) were calculated by Tukey's post-test (***P < 0.001, **P < 0.01 or *P < 0.05).

Furthermore, we further evaluate whether the structure of our molecular domino reactor would affect the efficacy of ^1^O_2_ production. Thanks to predesigned and desired DNA construction via automated modular synthesis technology, AS1411-0T-PA, AS1411-4T-PA, AS1411-10T-PA, AS1411-20T-PA and AS1411-40T-PA were synthesized precisely, showing various construction and distance between PA and G-quadruplex/hemin DNAzyme (**Figure 3A & Figure S8**). We studied the cancer cell-targeting capabilities of various AS1411-PA ApDCs using flow cytometry. MCF-7 cancer cells with high expression of nucleolin were incubated with AS1411-0T-PA, AS1411-4T-PA, AS1411-10T-PA, AS1411-20T-PA and AS1411-40T-PA for 1 h and then washed with washing buffer for removal of unbound materials. Subsequently, the targeting abilities of various AS1411-PA ApDCs were evaluated by flow cytometry **(Figure 3B)**. Since artificial base, PA, might block the formation of spatial configuration, AS1411-0T-PA demonstrated weak binding affinity towards MCF-7 cells, whereas AS1411-4T-PA, AS1411-10T-PA, AS1411-20T-PA and AS1411-40T-PA showed high binding affinities. Next, we further evaluated the efficacy of ^1^O_2_ production of AS1411/hemin-4T-PA, AS1411/hemin-10T-PA, AS1411/hemin-20T-PA, and AS1411/hemin-40T-PA in the presence of hydrogen peroxide under 670-nm NIR light irradiation for 10 min. As shown in **Figure 3C**, AS1411/hemin-4T-PA showed the highest light-induced ^1^O_2_ generation by decomposing inherent H_2_O_2_ to produce O_2_ and then utilizing the produced O_2_ to generate ^1^O_2_ in such confined DNA structure. This is due to AS1411/ hemin-4T-PA, a precise molecular domino reactor which offers the shortest distance between PA and G-quadruplex/hemin DNAzyme, allowing photosensitizer PA to efficiently utilize pre-produced O_2_ to generate ^1^O_2_. Thanks to these efficiently consecutive reactions, AS1411/hemin-4T-PA (abbreviated as AS1411/hemin-PA) can be considered as an ideal PDT agent, and then used in the further PDT *in vitro* and *in vivo*.

**Figure 4:**
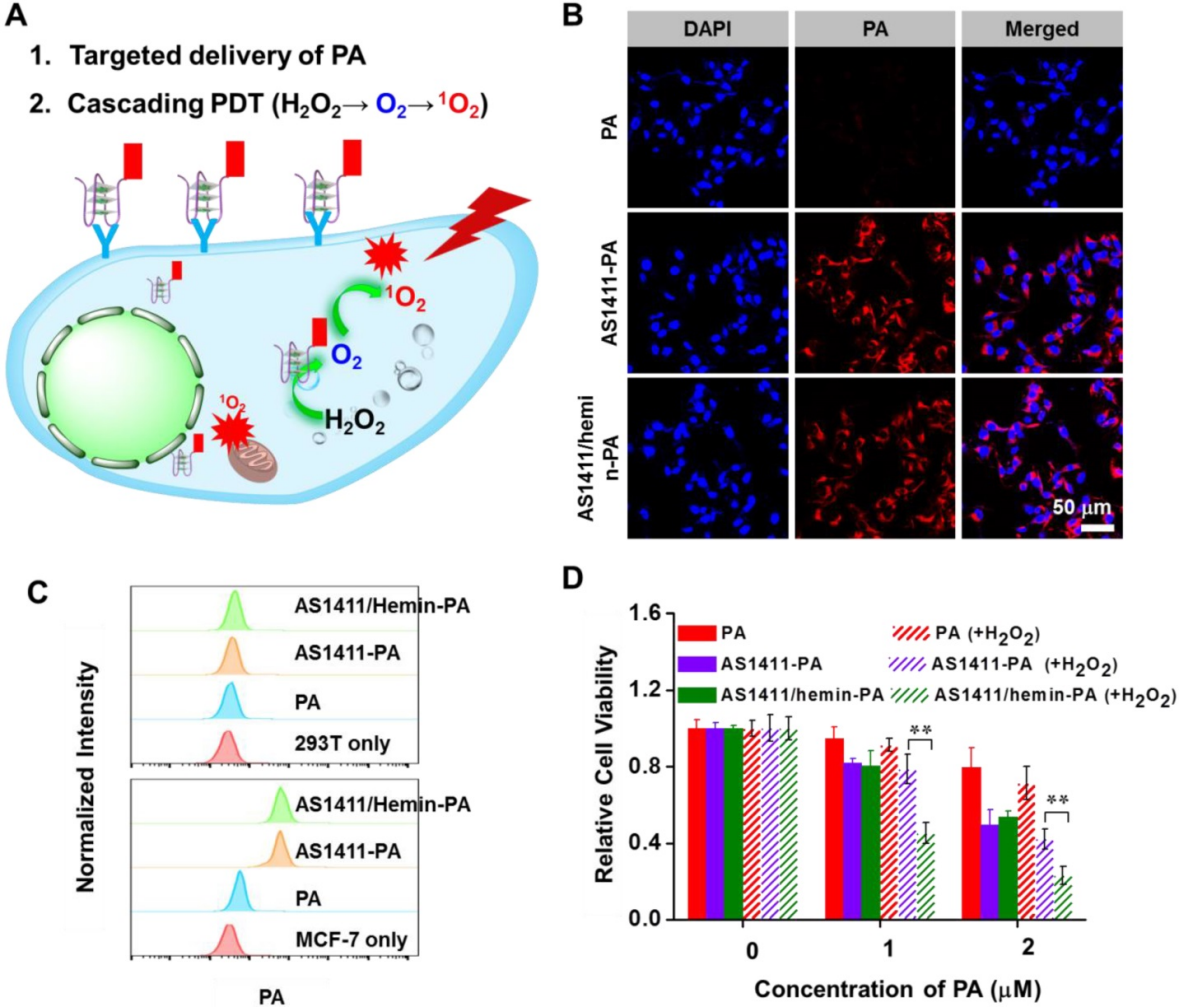
AS1411/hemin-PA for *in vitro* enhanced PDT. (A) Scheme illustrating targeted delivery of PA and cascading PDT. (B) Confocal images of MCF-7 cells incubated with free PA, AS1411-PA, or AS1411/hemin-PA with the same concentration of PA. Blue and red colors represent DAPI-stained cell nuclei and PA fluorescence, respectively. (C) Flow cytometric analysis of MCF-7 cells and 293T cells after treatment with 400 nM free PA, AS1411-PA or AS1411/hemin-PA in cell growth media for 1h. (D) *in vitro* PDT treatment of MCF-7 cells after treatment with free PA, AS1411-PA or AS1411/hemin-PA in the presence or absence of 100 μM H_2_O_2_ under 670-nm NIR light irradiation for 30 min at the power density of 5 mW/cm^2^. P values in (D) were calculated by Tukey's post-test (***P < 0.001, **P < 0.01 or *P < 0.05).

Next, the cancer cell-targeting capability of AS1411/hemin-PA was also measured using confocal imaging microscopy **(Figure 4B)**. Compared with free PA without obvious PA signals, both AS1411-PA- and AS1411/hemin-PA-treated MCF-7 cells showed the strong fluorescence signal of PA, indicating high target recognition of MCF-7 cells. Meanwhile, quantitative binding affinity was further determined by flow cytometry **(Figure 4C)**. For the non-targeted 293T cells, neither AS1411/hemin-PA nor AS1411-PA demonstrated obvious cell-targeting abilities. Whereas, for the targeted cancer cells, both AS1411/hemin- PA and AS1411-PA showed excellent selectivity toward target MCF-7 cells after incubation for 1 h, as compared with free PA.

Encouraged by the excellent target-binding ability of AS1411/hemin-PA, we further evaluated its targeted PDT efficiency *in vitro*. Without NIR light irradiation, AS1411/hemin-PA, AS1411-PA and free PA showed no toxicity to MCF-7 cells after incubation for 24h, even with high concentrations up to 20 μM, as revealed by 3-[4,5-dimethylthiazole-2-yl]-2,5-diphenyltetrazolium bromide (MTT) cell viability assay, indicating excellent biocompatibility of our AS1411/hemin-PA **(Figure S9)**. It is well known that cancer cells inside solid tumors can reduce the oxygen supply and produce upregulated levels of H_2_O_2_ owing to insufficient blood supply and aberrant metabolism 47, 48. Thus, MCF-7 cells were incubated with AS1411/hemin-PA, AS1411-PA and free PA under NIR light irradiation for 30 min without or with exogenous H_2_O_2_ (100 μM) used to mimic the unique TME. Owing to their specific targeting of MCF-7 cells, AS1411/hemin-PA and AS1411-PA showed highly effective cancer cell killing, as compared with free PA. In the presence of exogenous addition of H_2_O_2_, AS1411-PA showed comparable levels of PDT-induced cancer cell killing ability. Interestingly, the PDT efficiency of AS1411/hemin-PA was significantly enhanced by adding exogenous H_2_O_2_ based on the decomposition of H_2_O_2_ to produce oxygen and further generate singlet oxygen, achieving a domino of amplified PDT **(Figure 4D)**.

**Figure 5:**
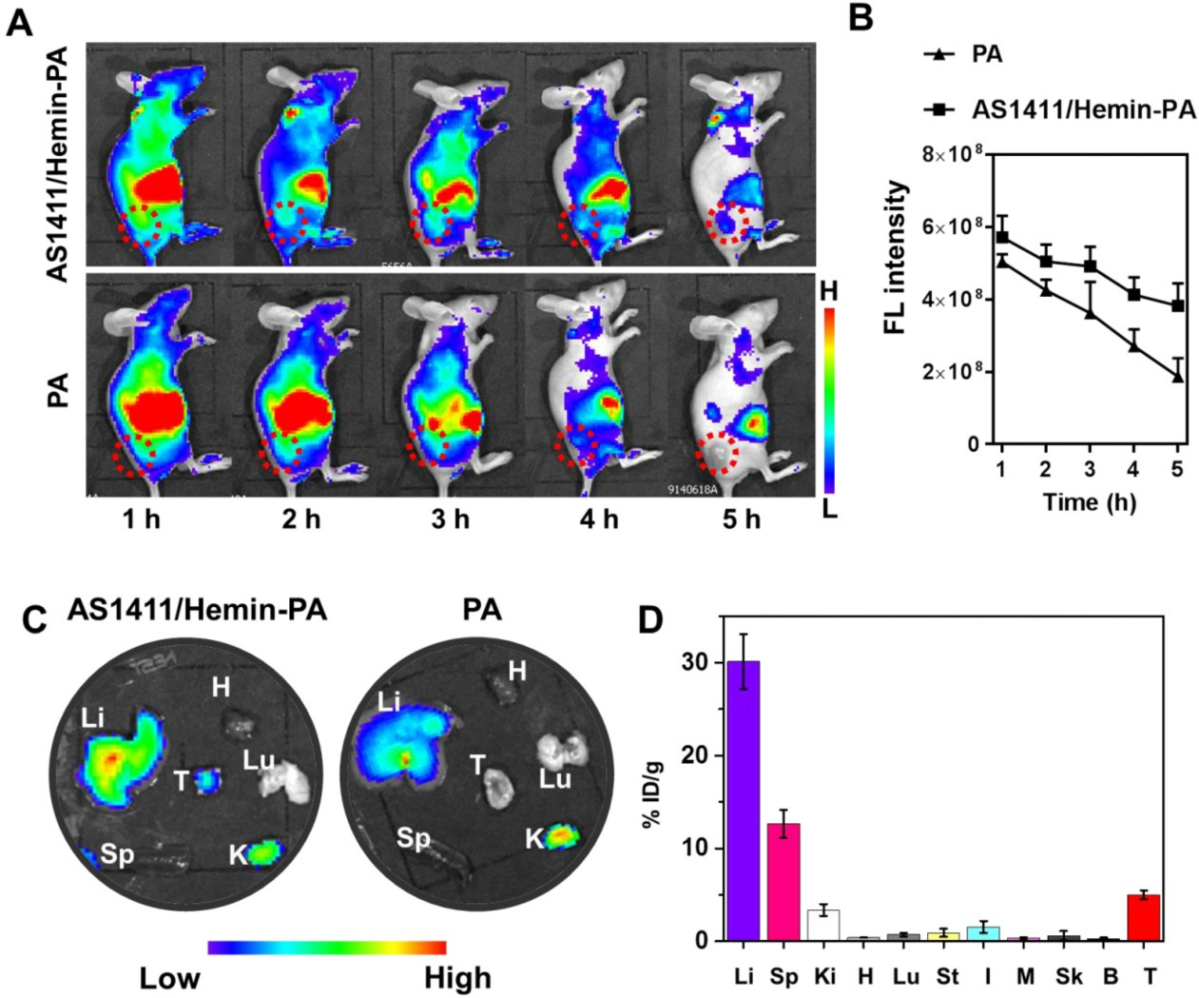
*In vivo* imaging and biodistribution of MCF-7-bearing mice after i.v. injection of AS1411/hemin-PA. (A) *In vivo* fluorescence imaging of mice bearing MCF-7 tumor at various time points post-injection of PA or AS1411/hemin-PA. (B) Relative fluorescence intensities of the tumors from different groups of mice at different time points based on *in vivo* fluorescence images shown in (A). (C) *Ex vivo* imaging of major organs and tumor extracted at 5 h post-injection of PA or AS1411/hemin-PA. (D) The biodistribution profiles of AS1411/hemin-PA in MCF-7-bearing mice at 5 h p.i. H, Li, Sp, Lu, Ki, St, I, M, Sk, B, and T stand for heart, liver, spleen, lung, kidneys, stomach, intestine, muscle, skin, bone, and tumor, respectively.

After investigating the *in vitro* PDT efficiency of AS1411/hemin-PA, we next studied its *in vivo* behavior. First, *in vivo* fluorescence imaging was employed to evaluate the targeted performance improvement of AS1411/hemin-PA for *in vivo* applications. After i.v. injection of free PA into nude mice bearing human MCF-7 tumor xenografts, no obvious PA fluorescence signal was detected at the tumor site. However, AS1411/hemin-PA, when intravenously injected, was first distributed throughout the entire body and then showed obvious tumor accumulation at the 3h post-injection (p.i.) point **(Figure 5A & 5B)**, indicating specific MCF-7 tumor-targeting recognition of AS1411/hemin-PA. Subsequently, at 5 h p.i., mice were sacrificed, and the tumor and major organs (liver, spleen, kidneys, heart, and lung) were collected and observed by *ex vivo* imaging. The PA signal in the tumor region of mice injected with AS1411/hemin-PA was much stronger than that of free PA-injected mice **(Figure 5C)**. Interestingly, mouse kidneys displayed obvious PA signals after injection of AS1411/hemin-PA, indicating significant renal filtration. To further analyze the *in vivo* behavior of AS1411/hemin-PA, MCF-7 tumor-bearing mice were sacrificed to harvest major organs 5 h post-injection of AS1411/hemin-PA. Next, the tissue distribution profile of AS1411/hemin-PA was determined by measuring PA fluorescence intensities. As presented in **Figure 5D**, AS1411/hemin-PA showed high accumulation in the liver by the Reticuloendothelial System (RES) responsible for the clearance of foreign molecules. Meanwhile, a relatively high fluorescence level was observed in the kidneys, suggesting the rapid clearance of AS1411/hemin-PA, which benefits biocompatibility and biodegradability. Importantly, AS1411/hemin-PA significantly accumulated in the tumor 5 h post-injection, as high as 5.2 percent of injected dose per-gram-tissue (%ID/g), showing that our AS1411/hemin-PA has promise for development as a candidate for cancer treatment.

**Figure 6:**
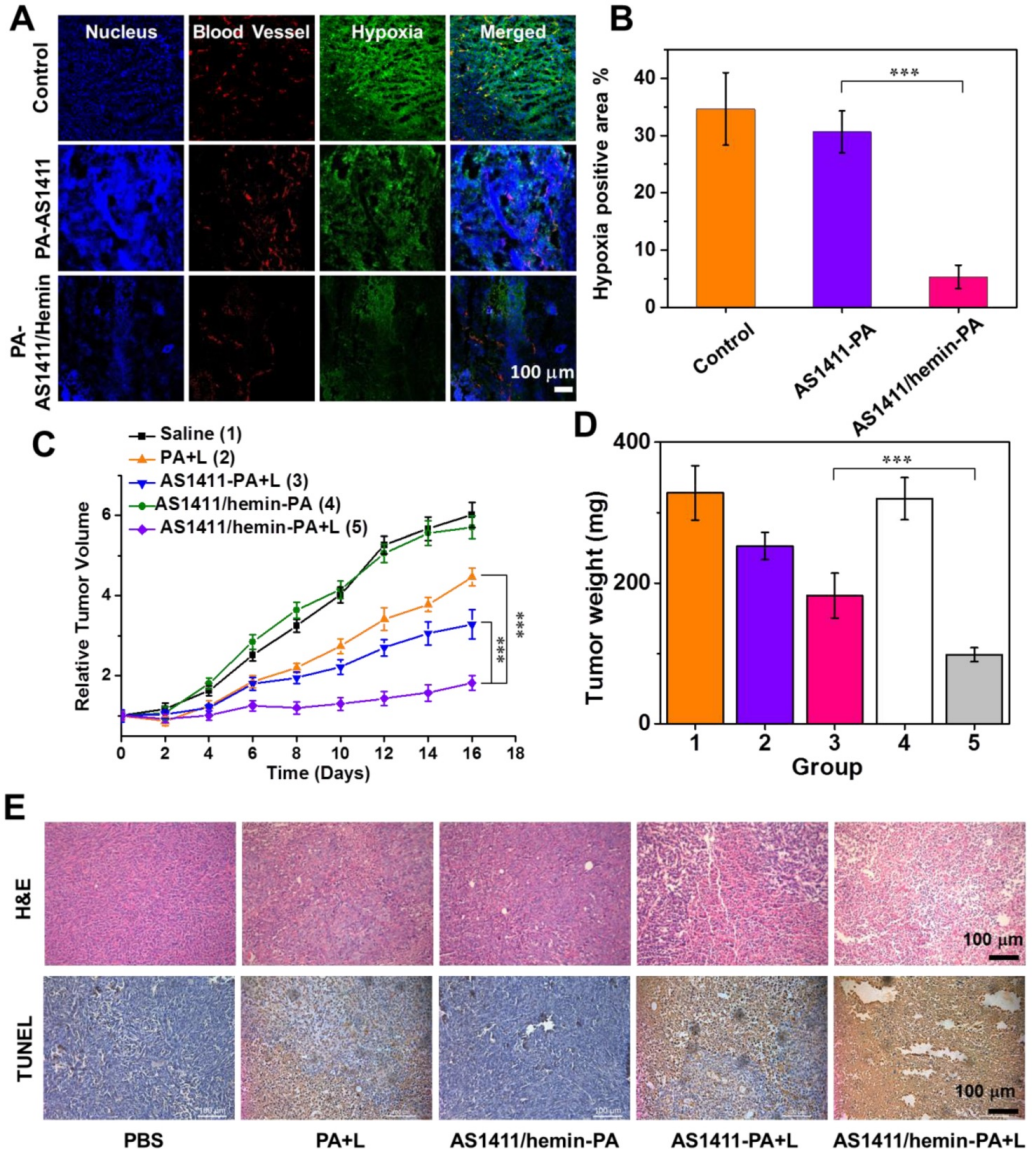
*In vivo* enhanced photodynamic therapy with AS1411/hemin-PA. (A) Representative immunofluorescence images of tumor slices extracted from mice treated with AS1411-PA or AS1411/hemin-PA after hypoxia staining. Blood vessels and hypoxic areas were stained by antiCD31 antibody (red) and anti-pimonidazole antibody (green), respectively. (B) Quantification of hypoxic areas in each group was recorded from more than 10 images using ImageJ software. (C) The tumor growth curves for various groups of mice (five mice per group) are indicated. (D) Average weights of mouse tumor in different groups at 16 days after various treatments. (E) Micrographs of H&E- and TUNEL-stained tumor slices extracted from various groups on the second day post-treatment. P values in (B,C,D) were calculated by Tukey's post-test (***P < 0.001, **P < 0.01 or *P < 0.05).

To confirm the ability of AS1411/hemin-PA to enhance oxygenation inside the solid tumor, immunofluorescence staining using a hypoxyprobe (pimonidazole hydrochloride)^49^ was performed on a frozen section of tumor extracted from mice 5 h post-injection with AS1411/hemin-PA. Cell nuclei, tumor blood vessels, and hypoxic areas, when imaged by confocal microscopy, were stained with DAPI (blue), anti-CD31 antibody (red) and anti- pimonidazole antibody (green), respectively **(Figure 6A)**. Compared with the control group, no obvious change in the green fluorescence was observed on the tumor slice treated with AS1411-PA. However, the AS1411/hemin-PA-treated group showed significantly reduced green pimonidazole-stained hypoxic fluorescence, suggesting efficient reduction of tumor hypoxia. The semiquantitative statistical analysis of hypoxia-positive areas from more than 10 confocal images for each group further confirmed that AS1411/hemin-PA, when intravenously injected, could suppress tumor hypoxia without affecting blood vessel densities **(Figure 6B)**. This reduction of tumor hypoxia in TME should sufficiently overcome hypoxia-associated photodynamic resistance during *in vivo* PDT.

Next, the amplified PDT therapeutic effect of AS1411/hemin-PA ApDC by utilization of the unique tumor microenvironment was evaluated by Balb/c mice bearing MCF-7 tumor. After MCF-7 tumors had grown to 60 mm^3^, 25 xenografted Balb/c mice were randomly divided into 5 groups of five mice each as follows: (1) control group i.v.-injected with saline, (2) free PA (dose: PA 1 mg/kg) injection plus NIR light irradiation, (3) AS1411-PA injection plus NIR light irradiation (dose: 1 mg/kg in terms of PA ), (4) AS1411/hemin-PA injection only (Dose: PA, 1 mg/kg; hemin, 2.79 mg/kg), (5) AS1411/hemin-PA injection plus NIR light irradiation (equivalent to group 4). At 5 h post-injection, the mice in groups 2, 3, and 5 were exposed to 670-nm NIR light irradiation for 60 min at a power density of 12 mW·cm^-2^. After receiving different treatments, the tumor sizes of various groups were tested using a digital caliper every 2 days over 16 days, and the tumor weights were recorded at day 16 **(Figure 6 C, D)**. Compared with the control group, group 2 injected with AS1411/hemin-PA displayed negligible influence on tumor growth. Meanwhile, PDT treatment of free PA showed moderate suppressive effect on tumor growth, whereas the group injected with AS1411-PA plus NIR light irradiation showed a reduction of tumor growth. Most importantly, the tumor growth of mice treated with AS1411/hemin-PA injection plus 670-nm light irradiation was remarkably inhibited, achieving an efficacy much better than that offered by all other groups **(Figure 6 C, D & Figure S11)**.

In addition, hematoxylin and eosin (H&E) staining and terminal deoxynucleotidyl transferase- mediated dUTP-biotin nick end labeling (TUNEL) assay were employed to further evaluate the PDT-triggered histological and pathological changes to tumors after various treatments **(Figure 6E)**. We observed that tumor treated with AS1411/hemin-PA plus NIR light irradiation displayed the most severe tumor cell damage with condensed nuclei and changed cell shapes, as revealed by H&E staining, whereas only moderate damage was found in mice treated with free PA plus NIR light and AS1411-PA plus NIR light. Furthermore, as shown in TUNEL staining, the most significant level of apoptosis was observed in the tumors of mice treated with AS1411/hemin-PA plus light, following the same trend as the results of H&E staining. During the entire treatment, mice with AS1411/hemin-PA injection plus NIR light demonstrated normal behavior and maintained stable body weights **(Figure S10)**. At day 16, major organs, including heart, liver, spleen, lung, and kidneys, were extracted for H&E staining to further evaluate the physiological toxicity of our AS1411/hemin-PA. As presented in **Figure S12**, major organs of mice in treatment group 5 with AS1411/hemin-PA plus light appeared as normal as those of the untreated mice, indicating the nontoxicity of our AS1411/hemin-PA.

## Conclusion

In summary, we developed an automated modular technology to prepare ApDCs as a molecular domino reactor for cancer treatment. The therapeutic phosphoramidite module (phosphoramidite PA) was synthesized and then incorporated into the aptamer using simple solid-phase fragment coupling methods. Utilizing AS1411 aptamer, which recognizes nucleolin on the cancer cell membrane, as a model, insertion of hemin into AS1411 G-quadruplex led to the decomposition of exogenous H_2_O_2_ into oxygen, releasing TME hypoxia-associated resistance. As evaluated by *in vitro* and* in vivo* studies, AS1411/hemin-PA ApDC as a molecular domino reactor, at the molecular level, could efficiently recognize cancer cells and utilize TME exogenous H_2_O_2_ to produce O_2_ and then further generate ^1^O_2_ for cascading amplified PDT. Overall, this modular technology provides a general strategy for creating functional ApDCs as a molecular domino reactor to overcome physiological barriers of the tumor microenvironment, thus promoting the future clinical translation of ApDCs.

## Experimental Procedures

### Materials

For organic synthesis, pyropheophorbide-A was obtained from Shanghai Xianhui Pharmaceutical Co. Ltd. All other reagents were purchased from Sigma-Aldrich and used without further purification. For DNA synthesis, bases and modifier reagents were purchased from Glen Research.

### Synthesis of pyropheophorbide-A phosphoramidites

#### Synthesis of compound 1

Pyropheophorbide (268 mg, 0.5 mmol), 1-ethyl-3(3-dimethylaminopropyl)-carbodiamide hydrochloride (115 mg, 0.6 mmol) and N-hydroxysuccinimide (69 mg, 0.6 mmol) were dissolved in 30 mL DCM. The dark solution was stirred overnight at 25 ^o^C. Next, 3-amino-1,2-propanediol (232 μL, 3 mmol) was added to the solution, and the resulting reaction mixture was stirred overnight at room temperature. The solvent was removed in vacuo, and the resulting residue was purified by flash column chromatography on silica gel (DCM/methanol = 10/1, v/v) to yield 1 as a black solid (249 mg, 82%). ^1^H NMR (400 MHz, Chloroform-d) δ 9.24 (s, 1H), 9.09 (d, J = 18.5 Hz, 1H), 8.50 (s, 1H), 7.89 (dd, J = 17.8, 11.7 Hz, 1H), 6.22 (d, J = 17.9 Hz, 1H), 6.11 (d, J = 11.5 Hz, 1H), 5.21 (d, J = 19.9 Hz, 1H), 4.96 (d, J = 19.8 Hz, 1H), 4.46 (d, J = 7.8 Hz, 1H), 4.22 (d, J = 7.3 Hz, 1H), 3.55 - 3.49 (m, 2H), 3.47 (s, 1H), 3.35 (s, 3H), 3.28 (d, J = 12.8 Hz, 3H), 3.21 (s, 1H), 3.15 (s, 3H), 2.68 - 2.54 (m, 2H), 2.40 - 2.32 (m, 2H), 2.10 (d, J = 10.1 Hz, 1H), 2.04 (s, 1H), 1.75 (d, J = 6.3 Hz, 3H), 1.58 (q, J = 6.9 Hz, 3H), 1.41 (d, J = 7.1 Hz, 2H), 0.89 (d, J = 6.3 Hz, 1H), -1.76 (s, 1H). HRMS (ESI+) calculated for C_36_H_41_N_5_O_4_: 608.3(M+H^+^); found: 608.2.

#### Synthesis of compound S1

A suspension of compound 1 (249 mg, 0.41 mmol), Li_2_CO_3_ (203 mg, 4.1 mmol) and DIPEA (715 uL, 4.1 mmol in anhydrous CH_2_Cl_2_ (40 mL)) was added to DMTBF_4_ (280 mg, 0.69 mmol) proportionately. CH_2_Cl_2_ (50 mL) was added to the reaction, and the diluted solution was washed with saturated saline solution and dried over anhydrous sodium sulfate. After removal of solvent, the residue was purified by flash column chromatography (ethyl acetate/petroleum ether/TEA = 80/20/2) giving compound S1 (212mg, 57%) as a black foam. ^1^H NMR (400 MHz, DMSO-d6) δ 9.56 (d, J = 3.9 Hz, 1H), 9.30 (d, J = 3.9 Hz, 1H), 8.86 (d, J = 3.1 Hz, 1H), 8.14 (ddd, J = 16.8, 11.7, 3.6 Hz, 1H), 7.80 (s, 1H), 7.32 (d, J = 8.0 Hz, 2H), 7.19 (s, 2H), 7.17 (s, 1H), 7.14 (t, J = 7.1 Hz, 1H), 6.98 (t, J = 8Hz, 1H), 6.87 (d, J = 6 Hz, 2H), 6.78 - 6.72 (m, 5H), 6.33 (d, J = 16.4 Hz, 1H), 6.16 (d, J = 11.6 Hz, 1H), 5.18 (d, J = 19.6 Hz, 1H), 5.04 (d, J = 19.6 Hz, 1H), 4.95 (s, 1H), 4.53 (d, J = 5.2 Hz, 2H), 4.23 (s, 1H), 3.71 (d, J = 8.4 Hz, 2H), 3.55 (d, J = 3.3 Hz, 3H), 3.52 (t, J = 3.6 Hz, 2H), 3.50 (s, 3H), 3.40 (d, J = 2.0 Hz, 3H), 3.11 (d, J = 3.8 Hz, 3H), 3.08 (d, J = 1.6 Hz, 2H), 3.06 (d, J = 2.0 Hz, 2H), 2.88 - 2.78 (m, 2H), 2.07 (d, J = 6.8 Hz, 2H), 1.76 (d, J = 20. Hz, 3H), 1.56 (td, J = 7.5, 3.2 Hz, 2H), 1.22 (d, J = 4.0 Hz, 1H), 0.98 (td, J = 7.1, 2.5 Hz, 2H), -2.09 (s, 1H). HRMS (ESI+) calculated for C_57_H_59_N_5_O_6_: 910.5(M+H^+^); found: 910.3.

#### Synthesis of compound 2 (phosphoramidite PA)

N,N-diisopropylchlorophosphoramidite (141 μL, 0.63 mmol) was added to 20 mL of anhydrous CH_2_Cl_2_ containing compound S1 (192 mg, 0.21 mmol) and N,N-Diisopropylethylamine (DIEA, 330 μL, 1.26mmol) at 0 ^o^C. After stirring at room temperature for 1 h, the solvent was removed in vacuo at room temprature. The residue was subjected to a flash silica gel column (ethyl acetate/petroleum ether/TEA, 20:80:2) to obtain compound 2 as a black foam (144 mg, 62 %). ^1^H NMR (400 MHz, Chloroform-d) δ 9.49 (d, J = 6.7 Hz, 1H), 9.39 (s, 1H), 8.58 (s, 1H), 8.01 (dd, J = 17.1, 12.2 Hz, 1H), 7.75 (s, 1H), 7.33 (d, J = 7.2 Hz, 2H), 7.25 - 7.21 (m, 3H), 7.12 (t, J = 6.8 Hz, 2H), 7.02 (q, J = 6.0 Hz, 1H), 6.71 (t, J = 7.3 Hz, 4H), 6.29 (d, J = 17.8 Hz, 1H), 6.17 (d, J = 13.4 Hz, 1H), 5.85 (d, J = 14.3 Hz, 1H), 5.25 (dd, J = 19.9, 8.5 Hz, 1H), 5.10 (dd, J = 20.0, 5.4 Hz, 1H), 4.52 (p, J = 7.6 Hz, 1H), 4.32 (d, J = 7.5 Hz, 1H), 4.27 - 4.17 (m, 1H), 4.17 - 4.08 (m, 1H), 3. 69 (q, J = 6.8 Hz, 2H), 3.66 (s, 6H), 3.64 (s, 3H), 3.59 - 3.46 (m, 3H), 3.40 (d, J = 7.9 Hz, 3H), 3.24 (s, 3H), 3.05 (t, J = 7.7 Hz, 1H), 2.95 (t, J = 7.8 Hz, 1H), 2.75 (dt, J = 6.4, 3.6 Hz, 2H), 2.32 (t, J = 11.5 Hz, 1H), 1.79 (t, J = 5.7 Hz, 3H), 1.70 (t, J = 8.0 Hz, 4H), 1.62 (s, 3H), 1.57 - 1.51 (m, 1H), 0.95 (d, J = 6.8 Hz, 6H), 0.92 (d, J = 7.0 Hz, 3H), 0.88 (d, J = 6.4 Hz, 3H), 0.45 (s, 1H), -1.71 (s, 1H). 13C NMR (101 MHz, Acetonitrile-d3) δ 196.26, 172.97, 172.74, 159.44, 159.39, 154.94, 150.76, 149.07, 146.02, 145.18, 141.82, 137.99, 136.34, 136.21, 135.82, 132.35, 130.87, 130.84, 129.79, 128.85, 128.59, 128.56, 128.26, 127.54, 127.52, 122.61, 119.34, 118.91, 113.86, 113.82, 104.17, 94.06, 61.56, 61.28, 59.16, 59.11, 55.65, 55.58, 45.99, 45.93, 45.79, 45.66, 41.52, 24.80, 24.72, 24.21, 24.15, 23.19, 23.17, 23.12, 23.10, 20.97, 20.88, 20.55, 17.60, 14.24, 12.30, 11.86, 10.92. ^31^P NMR (162 MHz, Acetonitrile-d3) δ 148.59. HRMS (ESI+) calculated for C_66_H_76_N_7_O_7_P: 1110.5(M+H^+^); found: 1110.3.

### Synthesis of AS1411-PA aptamer

AS1411-n(T)-PA (n= 0, 4, 10, 20, 40) aptamer with the sequence of 5'-PA- n(T) GG TGG TGG TGG TTG TGG TGG TGG TGG -3' was synthesized on a PolyGen C12 DNA/RNA solid-phase synthesizer. The final aptamer was deprotected by AMA solution (ammonium hydroxide/ 40% aqueous methylamine 1:1) for 30 min at 65 ^o^C. After purification by reversed-phase HPLC using a C-18 column, the product was incubated in 80% acetic acid for 30 min to remove 4,4'-dimethoxytrityl (DMT). Finally, the collected DNA product was dried for further usage.

To prepare the AS1411/hemin-PA ApDC, AS1411-PA aptamer dissolved in 100mM Tris-HCl buffer containing 10 mM KCl and 100 mM NaCl was heated at 90 ^o^C for 3 min and then cooled. Next, 3 equivalents of hemin pre-dispersed in DMSO were added and then stirred another 6 hours. After ultrafiltration with a molecular weight cutoff (MWCO) of 3K filters, the purified AS1411/hemin-PA ApDC was obtained. Subsequently, a UV-vis-NIR spectrometer (PerkinElmer Lambda 750) was employed to record the UV-vis-NIR absorption spectra, and the Zetasizer Nano-ZS (Malvern Instruments, UK) was employed to evaluate the dynamic size of AS1411/hemin-PA.

Measurement of singlet oxygen: AS1411-PA and AS1411/hemin-PA with or without the addition of 100 μM of hydrogen peroxide were irradiated under 670-nm light at a power density of 5 mW/cm^2^ for 30 min. To detect ^1^O_2_ generation, 2.5 mM of singlet oxygen sensor green (SOSG, Molecular Probes, USA) was added to the various samples. Next, the SpectraMax® i3x Platform was employed to evaluate the generated ^1^O_2_ by measuring the recovered fluorescence intensity of SOSG at 530 nm using an excitation wavelength of 494 nm. SOSG was also employed to detect ^1^O_2_ generation of AS1411/hemin- PA incubated in hydrogen peroxide without NIR light irradiation.

### Cellular experiments

Human breast MCF-7 cancer cells were cultured according to ATCC-recommended conditions in DMEM media containing 10% fetal bovine serum (FBS), 1% penicillin/streptomycin and 40U/mL insulin at 37 ^o^C under 5% CO_2_.

To investigate targeted drug delivery, 5×10^4^ MCF-7 cells seeded in 6-well plates until adherence to the plastic were incubated with free PA, AS1411-PA, and AS1411/hemin-PA. After 2h incubation at 37 ^o^C, the cells were washed twice with PBS and stained with DAPI for confocal imaging using a Leica SP5 laser scanning confocal microscope. Flow cytometry was also studied with a BD FACSCalibur™ platform using the same procedure by incubating cells in the binding buffer at 4 ^o^C for 30 min.

*In vitro* cytotoxicity and PDT efficacy were evaluated by viability tests using standard thiazolyl tetrazolium (MTT, Sigma Aldrich) assay. Briefly, human breast MCF-7 cells were seeded in a 96-well plate and incubated with various concentrations of free PA, AS1411-PA, and AS1411/hemin-PA for 24 h to test cell viability. To investigate *in vitro* PDT efficacy, MCF-7 cells were seeded in a 96-well plate and incubated with different concentrations of free PA, AS1411-PA, and AS1411/hemin-PA with or without 100 μM of hydrogen peroxide. Next, variously treated cells were exposed to 670-nm light irradiation at a power density of 5 mW/cm^2^ for 30 min. After culture in fresh DMEM cell culture medium for another 24 h, cell viability was measured using the MTT assay.

### *In vivo* imaging

Balb/c mice were obtained from Nanjing Peng Sheng Biological Technology Co Ltd. and used according to the approved procedure of the Soochow University Laboratory Animal Center. For establishment of an MCF-7 breast tumor model, 1×10^7^ MCF-7 cells suspended in 50 µL of serum-free PBS were subcutaneously injected into the backs of mice.

For *in vivo* imaging, mice bearing MCF-7 tumors were i.v.-injected with free PA and AS1411/ hemin-PA (1 mg/kg, in terms of PA). A Lumina III *in vivo* imaging system (PerkinElmer) was employed to observe *in vivo* fluorescence imaging at various time points (1h, 2h, 3h, 4h, 5h). At 5 h post-injection, major organs (liver, spleen, kidneys, heart, and lung) and tumor were collected from various treated mice for *ex vivo* imaging. Meanwhile, the major organs and tumor were homogenized and then dissolved in lysis buffer (1% sodium dodecyl sulfate (SDS), 1% Triton X-100, 40 mM Tris-acetate, 10 mM ethylenediaminetetraacetic acid (EDTA), and 10 mM dithiothreitol (DTT)) to analyze the fluorescence intensity of PA for biodistribution measurement.

Immunofluorescence staining: Tumor slices were obtained from mice bearing MCF-7 tumors at 5 h post-injection of AS1411-PA and AS1411/hemin-PA (dose: PA 1mg/kg, hemin 2.79 mg/kg). Thirty mg/kg of pimonidazole hydrochloride were intraperitoneally injected into mice 1.5 h before they were sacrificed to form stable adducts with proteins in the hypoxic environment. Anti-pimonidazole antibody (green), anti-CD31 antibody (red), and DAPI (blue) were employed to stain the hypoxic areas, blood vessels and cell nuclei, respectively, for confocal imaging, using a Leica SP5 confocal fluorescence microscope. Next, hypoxia positive areas (green) recorded from more than five micrographs for various groups were quantitatively analyzed via Image J.

### *In vivo* PDT treatment

For *in vivo* PDT treatment, mice bearing MCF-7 tumor were injected with AS1411/hemin-PA when the tumor volume reached 60 mm^3^. At 5 h post-injection, mice were irradiated by 670-nm NIR light for 60 min at a power density of 12 mW·cm^-2^. Briefly, this therapy experiment included five groups (5 mice per group): group 1, saline injection; group 2, 1 mg/kg of free PA injection plus 670-nm NIR irradiation for 60 min at a power density of 12 mW·cm^-2^; group 3, AS1411-PA injection plus NIR light irradiation (dose: 1 mg/kg in terms of PA ); group 4, AS1411/hemin-PA injection only (Dose: PA, 1 mg/kg; hemin, 2.79 mg/kg); group 5, AS1411/ hemin-PA injection plus NIR light irradiation (equivalent to group 4). On the 6^th^ day of treatment, subsequent treatments were conducted with the same processing parameters. The tumor sizes were measured by a digital caliper, and body weights of mice were recorded by electronic balance for 16 days. The tumor volume was calculated as volume = (tumor length) × (tumor width)^2^/2, and then the relative tumor volume was calculated as V/V_0_ (V_0_ was the initial volume). On the 4^th^ day of treatment, tumor tissues in each group were collected to obtain the slices for further TUNEL and H&E staining according to the manufacturers' protocols. On the 16^th^ day of treatment, the major organs were collected and then prepared for H&E staining, with major organs collected in healthy mice used as a control.

## Supplementary Material

Supplementary figures.
