# Supplementary material for: Molecular domino reactor built by automated modular synthesis for cancer treatment
Source: Theranostics. 2020 Mar 4;10(9):4030–41. doi: 10.7150/thno.43581 (PMC7086356; doi:10.7150/thno.43581)
Supplement: Supplementary file 1 — Supplementary figures. [file thnov10p4030s1.pdf]

Supporting Information

Molecular cascade reactor built by automated modular synthesis for cancer treatment

Yu Yang[#1,2,4], Jiaxuan He[#3], Wenjun Zhu[5], Xiaoshu Pan[4], Hoda Safari Yazd[4], Cheng Cui[3,4], Lu Yang[4], Xiaowei Li[4], Long Li[4], Liang Cheng[5], Liangzhu Feng[5], Ruowen Wang[1], Zhuang Liu[5]*, Meiwan Chen[2]*, Weihong Tan[1,3]*

1. Institute of Molecular Medicine (IMM), Renji Hospital, State Key Laboratory of Oncogenes and Related Genes, Shanghai Jiao Tong University School of Medicine, and College of Chemistry and Chemical Engineering, Shanghai Jiao Tong University, Shanghai 200240, China
2. State Key Laboratory of Quality Research in Chinese Medicine, Institute of Chinese Medical Sciences, University of Macau, Taipa, Macau, China
3. Molecular Science and Biomedicine Laboratory (MBL), State Key Laboratory of Chemo/Biosensing and Chemometrics, College of Chemistry and Chemical Engineering, College of Biology, Aptamer Engineering Center of Hunan Province, Hunan University Changsha, Hunan, 410082, China
4. Department of Chemistry, Department of Physiology and Functional Genomics, Center for Research at Bio/Nano Interface, Health Cancer Center, UF Genetics Institute, McKnight Brain Institute, University of Florida, Gainesville, Florida 32611-7200, USA
5. Institute of Functional Nano & Soft Materials Laboratory (FUNSOM), Soochow University, Suzhou, Jiangsu 215123, China

Yu Yang[#] and Jiaxuan He[#] contributed equally to this work.

Synthesis, purification, and characterization of oligonucleotides

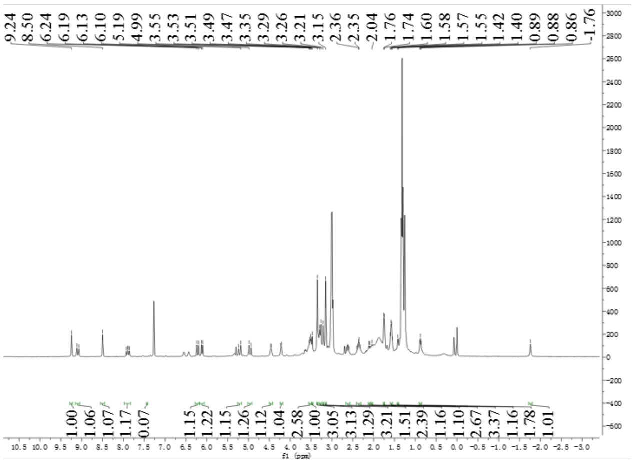

Figure S1 ^{1}H NMR spectrum of compound 1

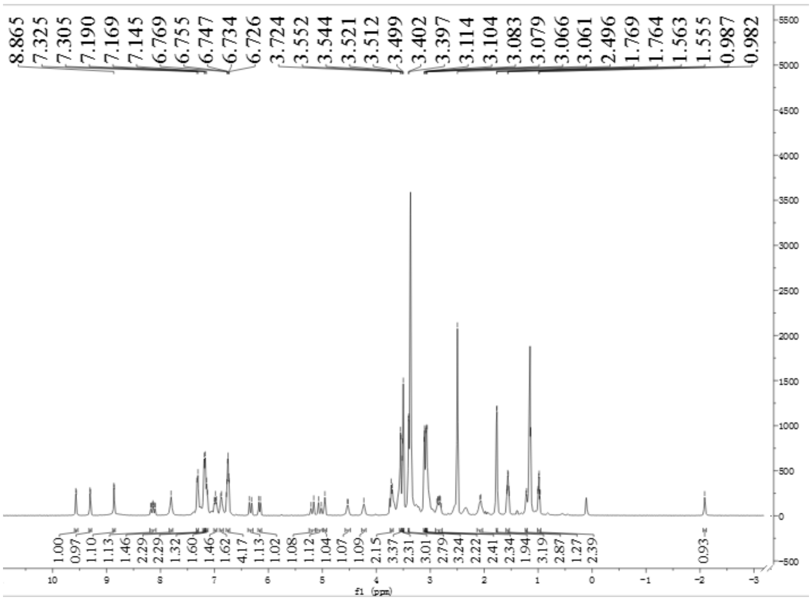

Figure S2 ^{1}H NMR spectrum of compound S1

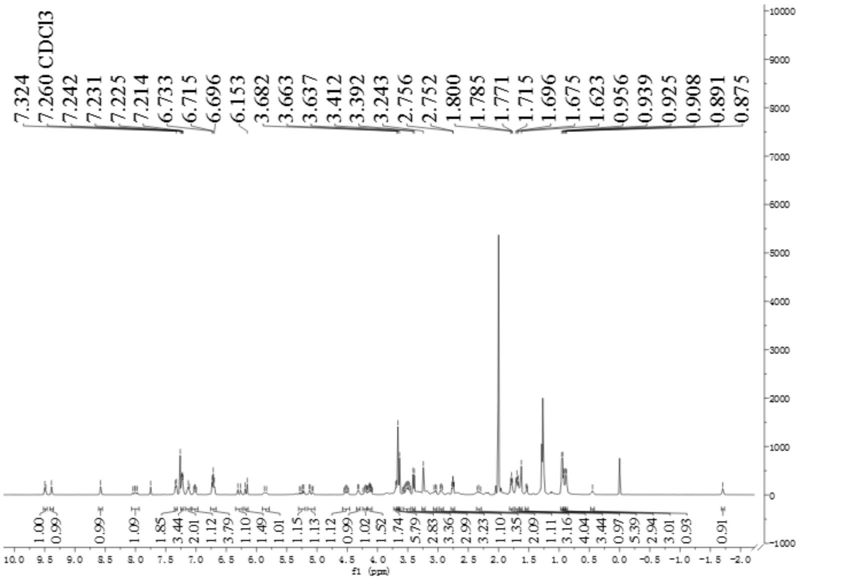

Figure S3 ^{1}H NMR spectrum of compound 2

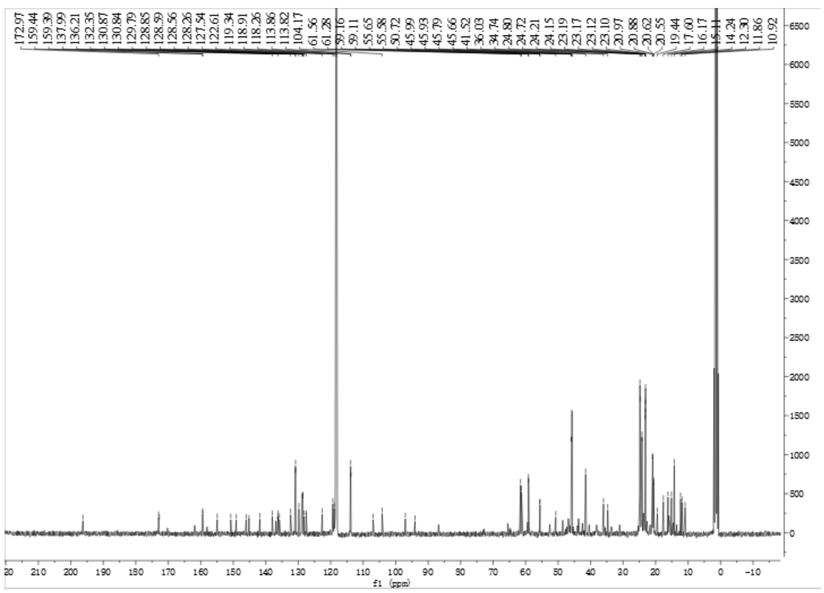

Figure S4 ^{13}C NMR spectrum of compound 2

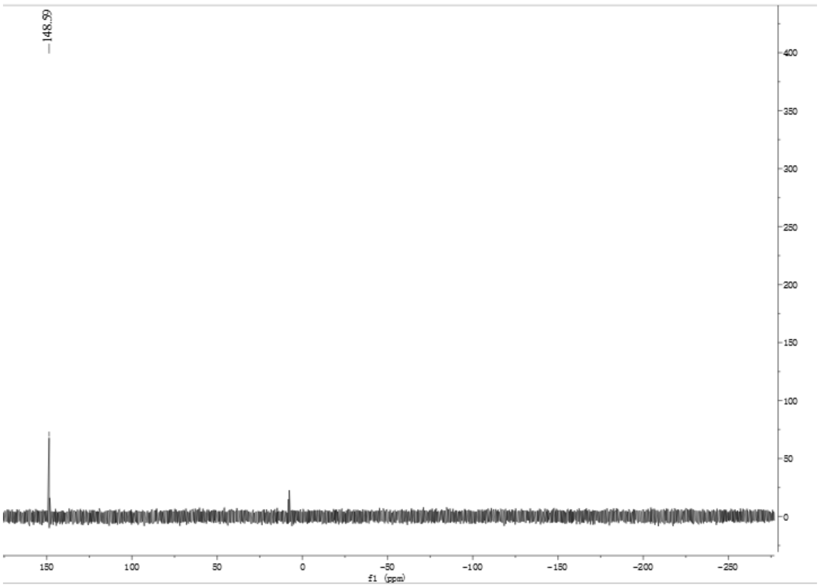

Figure S5 ^{31}P NMR spectrum of compound 2

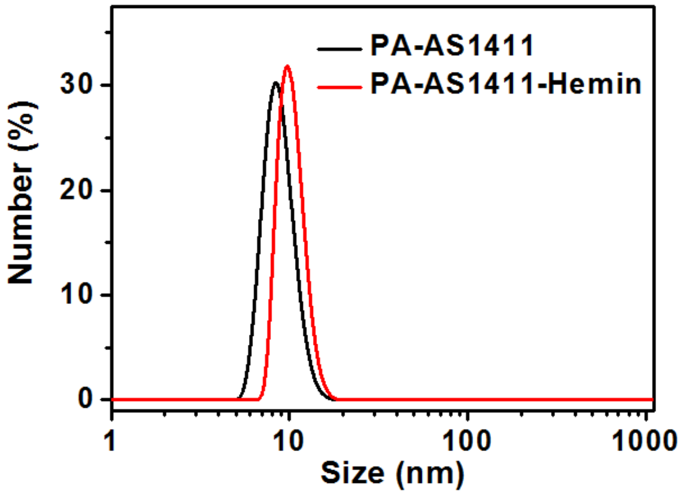

Figure S6 Hydrodynamic sizes of PAS1411-PA and AS1411/hemin-PA.

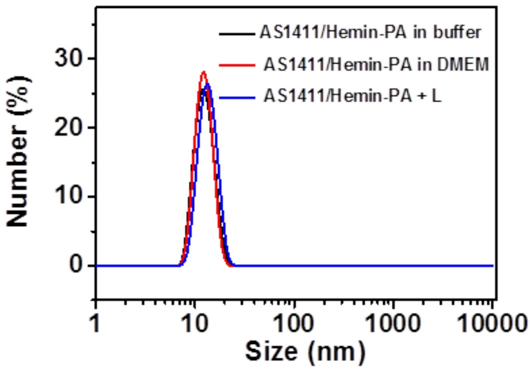

Figure S7 Hydrodynamic sizes of AS1411/hemin-PA in the various environments.

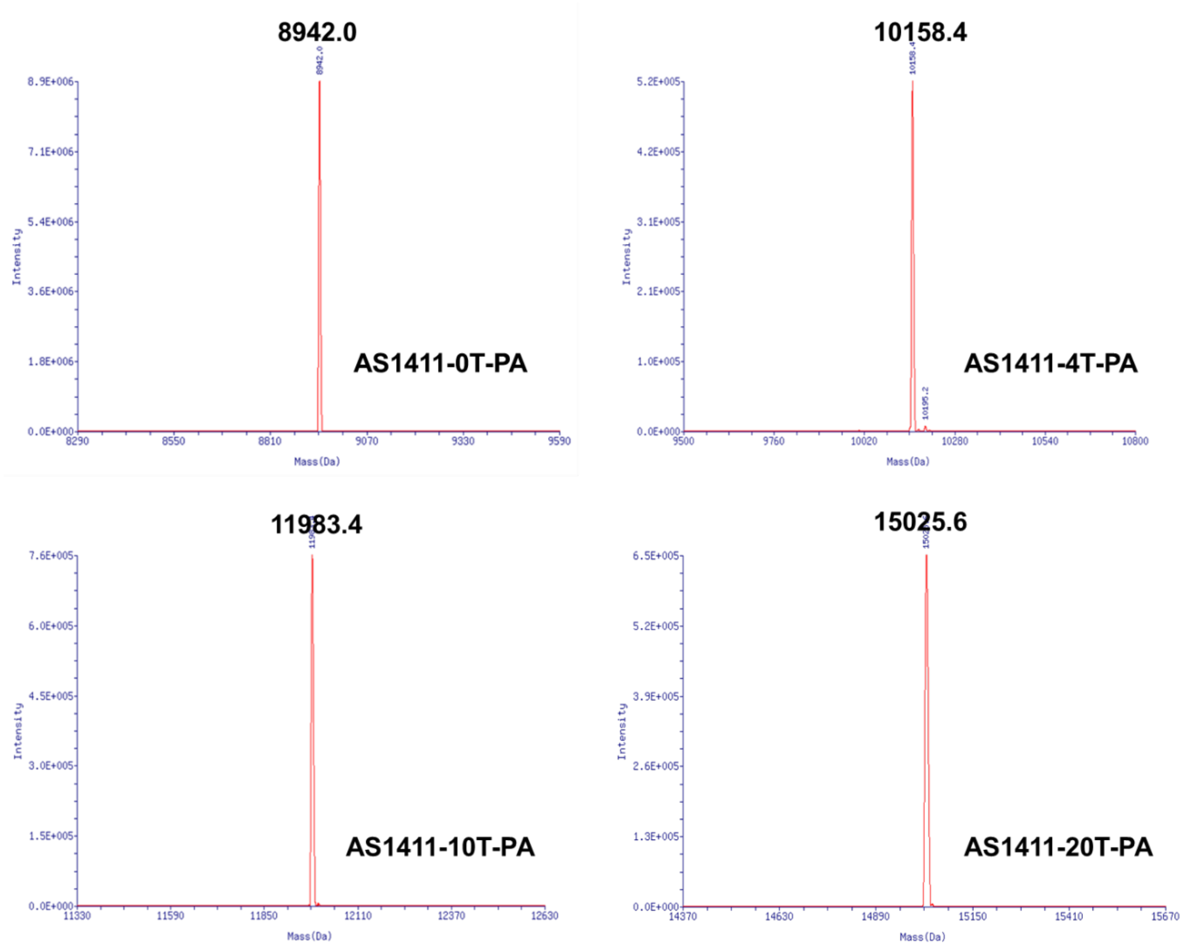

Figure S8 ESI-MS analysis of AS1411-0T-PA, AS1411-4T-PA, AS1411-10T-PA, AS1411-20T-PA by Sangon (Shanghai). Calculated molecular weights were 8941.64, 10158.42, 11983.6, 15025.56, and observed DNA peak was 8942.0, 10158.4, 11983.4, 15025.6 (M+H), respectively.

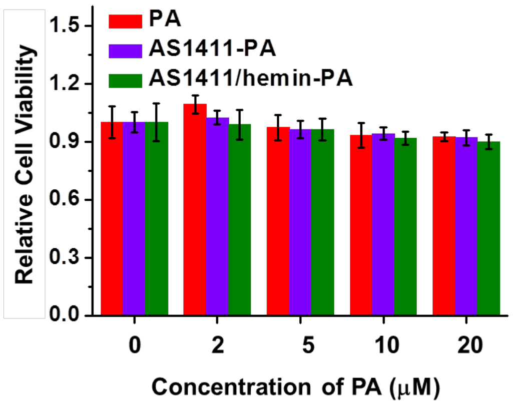

Figure S9 Cell viability of MCF-7 cells incubated with various concentrations of free PA, AS1411-PA or AS1411/hemin-PA in the dark for 24 h.

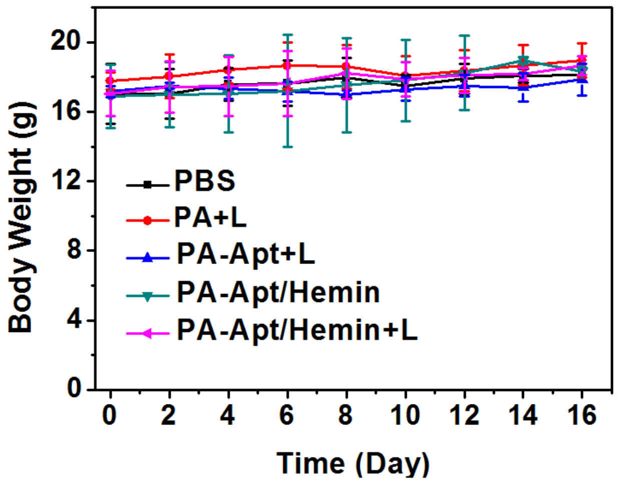

Figure S10 Body weight changes of various groups after treatment for 16 days.

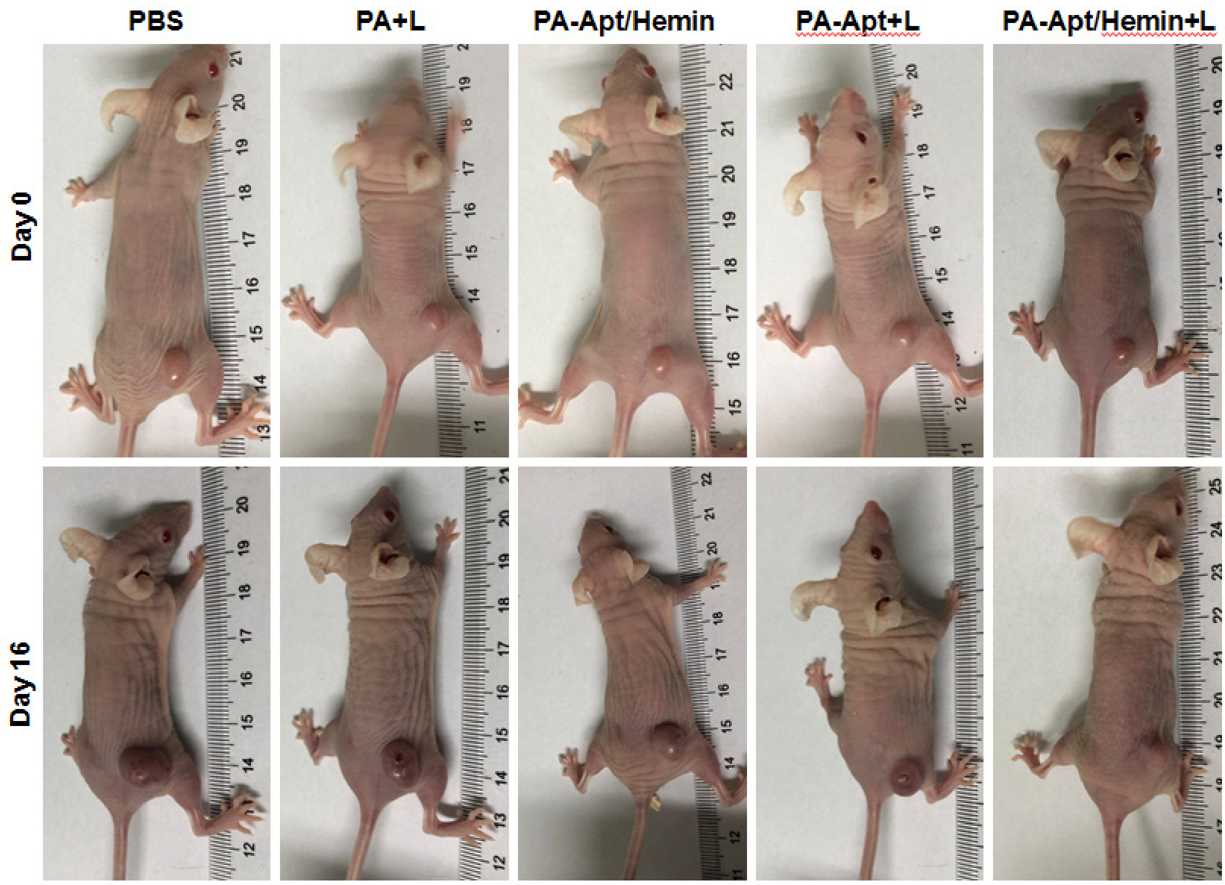

Figure S11 Photographs of mice after various treatments indicated at day 0 and day 16.

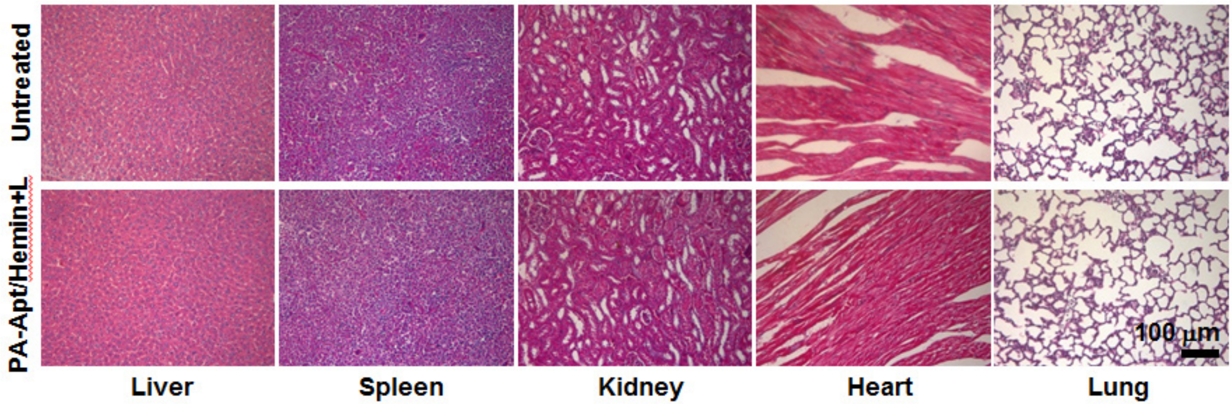

Figure S12 Microphotographs of H&E-stained major organs extracted from healthy mice or AS1411/hemin-PA plus light-treated mice.
